# PID, BFO-optimized PID, and PD-FLC control of a two-wheeled machine with two-direction handling mechanism: a comparative study

**DOI:** 10.1186/s40638-018-0089-3

**Published:** 2018-11-03

**Authors:** K. M. Goher, S. O. Fadlallah

**Affiliations:** 1 School of Engineering, University of Lincoln, Lincoln, UK; 2 Mechanical Engineering Department, Auckland University of Technology, Auckland, New Zealand

**Keywords:** Inverted pendulum, Two-wheeled machine, Two-direction handling, PID, BFO, FLC

## Abstract

In this paper; three control approaches are utilized in order to control the stability of a novel five-degrees-of-freedom two-wheeled robotic machine designed for industrial applications that demand a limited-space working environment. Proportional–integral–derivative (PID) control scheme, bacterial foraging optimization of PID control method, and fuzzy logic control method are applied to the wheeled machine to obtain the optimum control strategy that provides the best system stabilization performance. According to simulation results, considering multiple motion scenarios, the PID controller optimized by bacterial foraging optimization method outperformed the other two control methods in terms of minimum overshoot, rise time, and applied input forces.

## Introduction

For a tremendous amount of research studies, providing the ideal control strategy for inverted pendulum (IP)-based systems has been and still remains a field of interest. This can be related to the incomparable increase in the two-wheeled machines (TWMs) that serves nowadays in many applications, especially in applications that demand working in bounded spaces. For these types of highly unstable nonlinear systems, divergent control approaches have been established [1]. Some of these control methods include proportional–integral–derivative (PID) control scheme, bacterial foraging optimization (BFO) of PID control method, and fuzzy logic control (FLC) method.

### Proportional–integral–derivative (PID) control method

This control loop feedback mechanism has been commonly utilized in various control systems, specifically in systems that are based on the inverted pendulum principle. Ren et al. [2] presented a motion control and stability analysis study of a two-wheeled vehicle (TWV). For providing a motion control system that balances the TWV and enables the vehicle to track a predefined path, a self-tuning PID control strategy is proposed. By employing the same PID control approach with an observer-based state feedback control algorithm, Olivares and Albertos [3] presented and controlled an under-actuated flywheel IP system. The study conducted by Wang [4] addressed in detail the issue of adjusting multiple PID controllers simultaneously for the purpose of stabilization and tracking control of three types of IPs.

### Bacterial foraging optimization (BFO) algorithm

Initiated by Passino [5], bacterial foraging optimization (BFO) algorithm has been utilized in multiple research aspects and in different applications. Kalaam et al. [6] implemented BFO algorithm in a cascaded control scheme designed for controlling a grid-connected photovoltaic system. For modeling a single-link flexible manipulator system, Supriyono and Tokhi [7] developed an adaptable chemotactic step size bacterial foraging optimization (BFO) technique. Almeshal et al. [8] utilized the BFO algorithm on a smart fuzzy logic control scheme applied on a unicycle class of differential drive robot on irregular rough terrain.

Significant research studies focused on improving the BFO algorithm’s performance. These improvements were achieved either by combining BFO with another optimization approach [9, 10] or by modifying the algorithm’s actual parameters [11].

Focusing on IP-based systems, Agouri et al. [12] developed a control scheme based on quadratic adaptive bacterial foraging algorithm (QABFA) for controlling a two-wheeled robot with an extendable intermediate body (IB) moving on an inclined surface. Al-rashid et al. [13] applied a constrained adaptive bacterial foraging optimization strategy for optimizing the control gains of a single-link inverted pendulum on cart system. On the other hand, Jain et al. [14] implemented BFO algorithm in tuning a PID controller utilized in controlling an inverted pendulum system on field-programmable gate array (FPGA).

### Fuzzy logic control (FLC) method

Although the concept of fuzzy logic controller (FLC) was initiated in the 1960s [15], tremendous research studies applied this type of control scheme on IP-based systems because of its ability to deal with nonlinear systems, not to mention its intuitive nature. Czogała et al. [16] presented a rough fuzzy logic controller for stabilizing a pendulum-car system. As for Cheng et al. [17], their study focused on developing a FLC, with a high accuracy and resolution, for the purpose of stabilizing a double IP. On the other hand, Xu et al. [18] designed a FLC which obtains fuzzy rules from a simplified lookup table to stabilize a two-wheeled inverted pendulum. For the same aim, Azizan et al. [19] proposed a smart fuzzy control scheme for two-wheeled human transporter. The applied control method, when tested against different mass values that represent the transporter’s rider, revealed a high robustness. For an under-actuated two-wheeled inverted pendulum vehicle with an unstable suspension that is subjected to non-holonomic constraint, Yue et al. [20] developed a composite control approach that consists of a direct fuzzy controller and an adaptive sliding mode technique. Amir et al. [21], for an IP on a cart, developed an effective hybrid swing-up and stabilization controller (HSSC) that consists of three controllers: swing-up controller, fuzzy stabilization controller, and fuzzy switching controller. As for Yue et al. [22], their study aimed to develop an indirect adaptive fuzzy control that is based on an error data-based trajectory planner for controlling a wheeled inverted pendulum vehicle. Other research studies, such as Tinkir et al. [23], focused on comparing a conventional PID controller and an interval type 2 fuzzy logic (IT2FL) control method in order to control the swing-up position of a double IP.

### Research objective and paper organization

In order to provide the optimal control strategy for IP-based machines and to improve their stability performance, this paper sets a comparison between three control methods: PID controller, bacterial foraging optimization of PID controller, and fuzzy logic controller applied to control and stabilize a five-degrees-of-freedom (DOF) two-wheeled robotic machine (TWRM) introduced by Goher [24]. Despite the tremendous amount of control methods, the potential of the three selected approaches when it comes to dealing with highly unstable nonlinear systems such as inverted pendulums, as demonstrated in the literature, has encouraged the authors to investigate their implantation on the new five-DOF TWRM. The developed five-DOF two-wheeled machine, compared to current TWRMs, delivers payload handling in two mutually perpendicular directions while attached to the intermediate body (IB). This feature, as a result, increases the vehicle’s flexibility and workspace and permits the employment of TWRMs in service and industrial robotic applications (i.e., material handling, objects assembly). The rest of the paper is organized as follows: "Two-wheeled robotic machine system description" section demonstrates a detailed description of the five-DOF two-wheeled machine that the control approaches were implemented on. The system’s derived mathematical model is presented in "TWRM mathematical modeling" section. As for "Control system design" section, it illustrates the control system design and the implementation of the three control methods: PID controller, bacterial foraging optimization of PID controller, and fuzzy logic controller on the TWRM’s derived mathematical model. "Conclusions" section concludes the paper by highlighting the findings of the research.

## Two-wheeled robotic machine system description

**Fig. 1:**
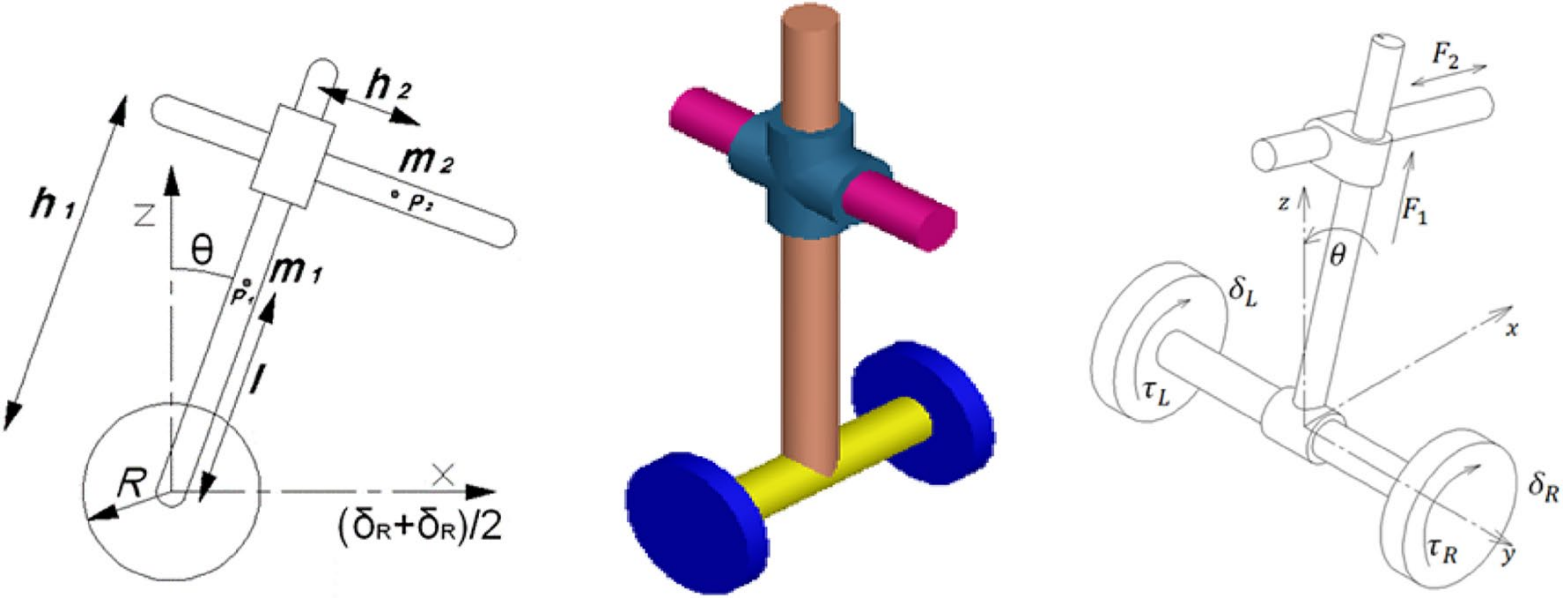
Two-wheeled robotic machine schematics diagram

The schematics diagram of the developed two-wheeled robotic machine (TWRM) is illustrated in Fig. 1. The robotic system consists of chassis, with center of gravity at point *P*_1_, and the linear actuators’ mass, with center of gravity at point *P*_2_. As long as the wheeled machine maneuvers far from its initial position, along the *X*-axis, *P*_1_ and *P*_2_ coordinates will vary. Each wheel has been connected to a motor that provides the substantial torque, *τ*_R_ and *τ*_L_, needed to control the TWRM. Both accelerometer and gyroscope sensors were fit to the robotic system in order to provide the necessary state variables that enables the applied control scheme to preserve the TWRM’s position at the upright position uninterruptedly. With respect to the *X*- and *Z*-axis and referring to Fig. 1, the TWRM’s five DOFs can be defined as the following:

- The attached payload linear displacement in vertical direction (*h*_1_).
- The attached payload linear displacement in horizontal direction (*h*_2_).
- The angular displacement of the angular rotation of the right wheel (*δ*_R_).
- The angular displacement of the angular rotation of the left wheel (*δ*_L_).
- The tilt angle of the intermediate body around the vertical *Z*-axis (*θ*).

For a picking and placing scenario, Table 1 demonstrates the engagement of each of the wheeled machine’s actuators for each sub-task, along with the DOFs associated with the corresponding process task. The reason behind the continuous activation of the TWRM wheels’ motors is due to the external disturbances taking place while performing the picking and/or placing task, as well as the center of mass’s continuous variation. Therefore, it is crucial to the wheels’ motors to develop the necessary torque signal in order to maintain the upright vertical position of the TWRM. Moving to the linear actuators, their engagement is related to the appointed sub-task. Switching mechanisms are designed, as a major part of the three investigated control schemes, in order to define the period of engagement of each individual actuator in service.

**Table 1:**
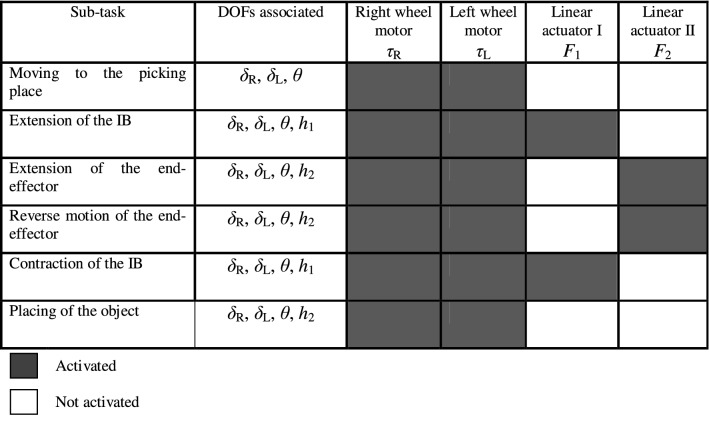
Engagement of individual actuators for each sub-task [24]

## TWRM mathematical modeling

The TWRM’s mathematical model, explained in detail by Goher [24], is derived by employing Lagrangian modeling approach, which is considered as one of the powerful techniques for obtaining the equations of motion for any sophisticated system. Referring to the two-wheeled robotic machine’s schematics diagram in Fig. 1 and its physical parametric specifications listed in Table 2, the system’s kinematics was related to the torques/forces applied to its links and the five highly coupled differential equations of motion are represented as follows:

**Table 2:** TWRM parameters description [24]

Terminology	Description	Value	Unit
*θ*	Tilt angle of the intermediate body around the vertical *Z-*axis	–	°
*δ*_R_ *, δ*_L_	Angular displacement of right and left wheels	–	m
*h*_1_, *h*_2_	Vertical and horizontal linear link displacement	–	m
*F*_1_, *F*_2_	Force generated by the vertical and horizontal linear actuators	–	N
*τ*_R_ *, τ*_L_	Right and left wheels torque	–	N/m
*m*_1_	Mass of the chassis	3.1	kg
*m*_2_	Mass of the linear actuators	0.6	kg
*m*_w_	Mass of wheel	0.14	kg
*R*	Wheel radius	0.05	m
*J*_1_	Chassis moment of inertia	0.068	kg m^2^
*J*_2_	Moving mass moment of inertia	0.0093	kg m^2^
*J*_w_	Wheel moment of inertia	0.000175	kg m^2^
*Ɩ*	Distance of chassis’ center of mass for wheel axle	0.14	m
*µ*_1_	Coefficient of friction of vertical linear actuator	0.3	Ns/m
*µ*_2_	Coefficient of friction of horizontal linear actuator	0.3	Ns/m
*µ*_w_	Coefficient of friction between wheel and ground	0	Ns/m
*µ*_c_	Coefficient of friction between chassis and wheel	0.1	Ns/m
*g*	Gravitational acceleration	9.81	m/s^2^

1 $$\begin{aligned} & 2m_{2} \dot{\theta }\left( {\dot{h}_{2} h_{2} + \dot{h}_{1} h_{1} } \right) + \tfrac{1}{2}m_{2} (h_{1} \cos \theta - h_{2} \sin \theta )\left( {\ddot{\delta }_{\text{R}} + \ddot{\delta }_{\text{L}} } \right) + \tfrac{1}{2}m_{1} l\cos \theta \left( {\ddot{\delta }_{\text{R}} + \ddot{\delta }_{\text{L}} } \right) \\ & \quad - m_{2} g(h_{1} \sin \theta + h_{2} \cos \theta ) + \ddot{\theta }\left( {J_{1} + J_{2} + m_{1} l^{2} + m_{2} h_{2}^{2} + m_{2} h_{1}^{2} } \right) \\ & \quad + m_{2} \left( {\ddot{h}_{2} h_{1} + \ddot{h}_{1} h_{2} } \right) - m_{1} gl\sin \theta = 0 \, \\ \end{aligned}$$

2 $$\begin{aligned} & \tfrac{1}{2}m_{1} \left( {\tfrac{1}{2}\ddot{\delta }_{\text{R}} + \tfrac{1}{2}\ddot{\delta }_{\text{L}} - l\dot{\theta }^{2} \sin \theta + l\ddot{\theta }\cos \theta } \right) + \tfrac{1}{2}m_{2} \left( {\ddot{h}_{1} \sin \theta + 2\dot{h}_{1} \dot{\theta }\cos \theta - h_{1} \dot{\theta }^{2} \sin \theta + h_{1} \ddot{\theta }\cos \theta } \right. \\ & \quad \left. { + \,\ddot{h}_{2} \cos \theta - 2\dot{h}_{2} \dot{\theta }\sin \theta - h_{2} \dot{\theta }^{2} \cos \theta - h_{2} \ddot{\theta }\sin \theta + \tfrac{1}{2}\ddot{\delta }_{\text{R}} + \tfrac{1}{2}\ddot{\delta }_{\text{L}} } \right) \\ & \quad + 2m_{\text{w}} \ddot{\delta }_{\text{R}} + 2J_{\text{w}} \frac{{\ddot{\delta }_{\text{R}} }}{{R^{2} }} = \tau_{\text{R}} - \mu_{\text{w}} \left( {\frac{{\dot{\delta }_{\text{R}} }}{{R^{2} }}} \right) - \mu_{\text{c}} \dot{\delta }_{\text{R}}^{{}} \\ \end{aligned}$$

3 $$\begin{aligned} & \tfrac{1}{2}m_{1} \left( {\tfrac{1}{2}\ddot{\delta }_{\text{R}} + \tfrac{1}{2}\ddot{\delta }_{\text{L}} - l\dot{\theta }^{2} \sin \theta + l\ddot{\theta }\cos \theta } \right) + \tfrac{1}{2}m_{2} \left( {\ddot{h}_{1} \sin \theta + 2\dot{h}_{1} \dot{\theta }\cos \theta - h_{1} \dot{\theta }^{2} \sin \theta + h_{1} \ddot{\theta }\cos \theta } \right. \\ & \quad \left. { + \,\ddot{h}_{2} \cos \theta - 2\dot{h}_{2} \dot{\theta }\sin \theta - h_{2} \dot{\theta }^{2} \cos \theta - h_{2} \ddot{\theta }\sin \theta + \tfrac{1}{2}\ddot{\delta }_{\text{R}} + \tfrac{1}{2}\ddot{\delta }_{\text{L}} } \right) \\ & \quad + 2m_{\text{w}} \ddot{\delta }_{\text{L}} + 2J_{\text{w}} \frac{{\ddot{\delta }_{\text{L}} }}{{R^{2} }} = \tau_{\text{L}} - \mu_{\text{w}} \left( {\frac{{\dot{\delta }_{\text{L}} }}{{R^{2} }}} \right) - \mu_{\text{c}} \dot{\delta }_{\text{L}}^{{}} \\ \end{aligned}$$

4 $$\tfrac{1}{2}m_{2} \left( {2g\cos \theta - 2h_{1} \dot{\theta }^{2} - 4\dot{h}_{2} \dot{\theta } - 2h_{2} \ddot{\theta } + 2\ddot{h}_{1} + (\ddot{\delta }_{\text{R}} + \ddot{\delta }_{\text{L}} )\sin \theta } \right) = F_{1} - \mu_{1} \dot{h}_{1}$$

5 $$\tfrac{1}{2}m_{2} \left( {2g\sin \theta + 2h_{2} \dot{\theta }^{2} - 4\dot{h}_{1} \dot{\theta } - 2h_{1} \ddot{\theta } - 2\ddot{h}_{2} - (\ddot{\delta }_{\text{R}} + \ddot{\delta }_{\text{L}} )\cos \theta } \right) = F_{2} - \mu_{2} \dot{h}_{2}$$

**Fig. 2:**
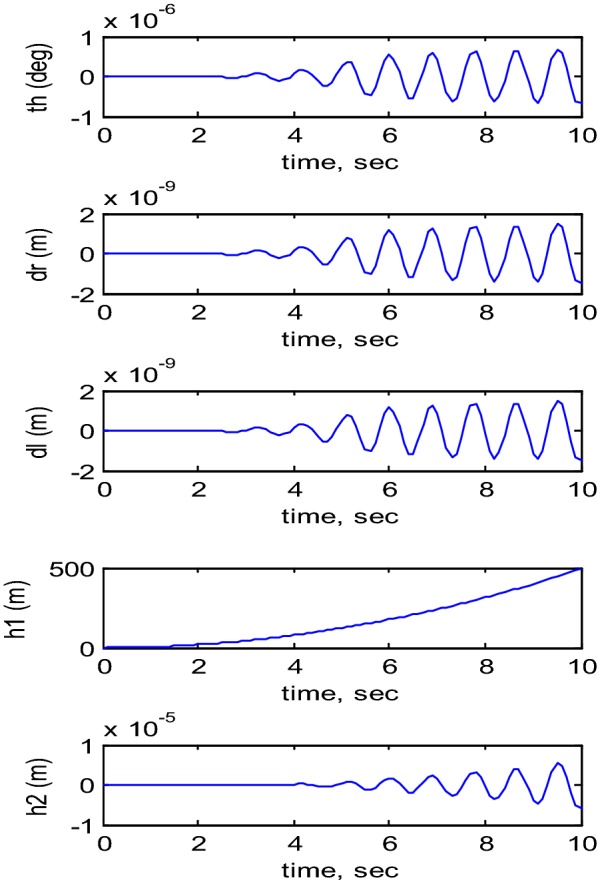
Open-loop system response [24]

The developed mathematical model of the TWRM, considering the simulation parameters listed in Table 2, is simulated in MATLAB/Simulink^®^ environment, and an open-loop response investigation was carried out in order to examine the behavior of the developed model. Figure 2 illustrates the system’s open-loop simulation results. It is clear from the simulation results of the five targeted control variables [i.e., pitch angle (*θ*), vertical link displacement (*h*_1_), horizontal link displacement (*h*_2_), right wheel displacement (*δ*_R_), and left wheel displacement (*δ*_L_)] that the TWRM is a nonlinear unstable system that requires a closed-loop configuration in order to achieve the desired performance in terms of stabilizing the TWRM.

## Control system design

This section concentrates on implementing and comparing the three control strategies (i.e., PID, bacterial foraging optimization of PID, and fuzzy logic control) for the sake of providing the optimal control strategy that improves the stability performance of the five-DOF TWRM by controlling the system’s main variables [i.e., angle of the robot’s chassis (*θ*), angular position of the right wheel (*δ*_R_), angular position of the left wheel (*δ*_L_), linear displacement of the attached payload in vertical direction (*h*_1_), linear displacements of the attached payload in horizontal direction (*h*_2_)].

### PID control design

**Fig. 3:**
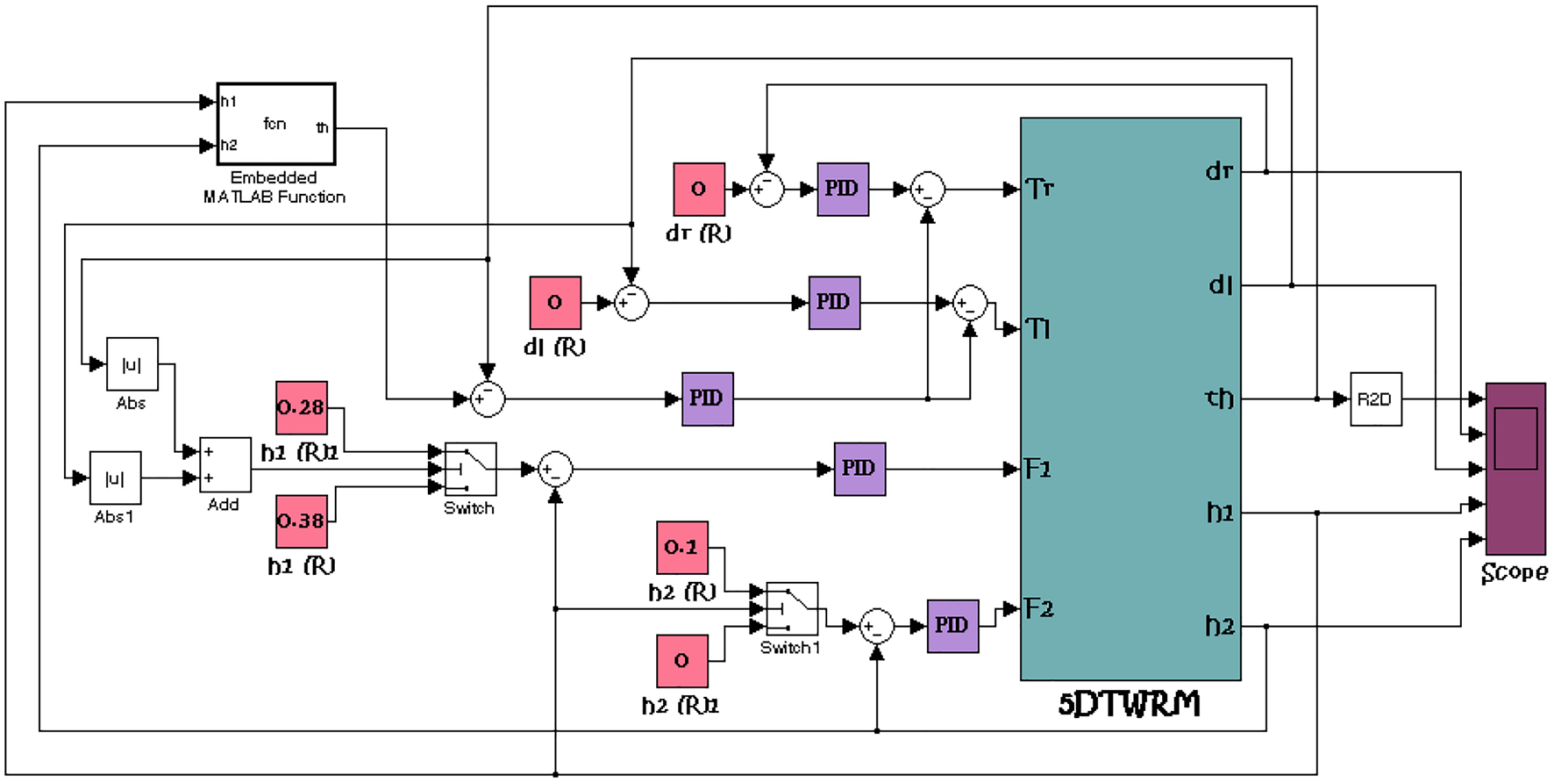
Simulink model of the PID controller implementation [24]

The strategy schematics which are based on designing a feedback control mechanism mainly consist of five control loops, for controlling the TWRM by employing PID control scheme which is demonstrated in Fig. 3. By measuring the error in the tilt angle of the IB, the angular position of the IB is controlled. Out of the five feedback control loops, two are designed in order to control the position of the object by considering the object position’s error as an input and the actuation force as an output. As for the two remaining control loops, they are designed with a view to mobilize the TWRM to follow a certain planner motion in the *XY* plane. For these two feedback loops, the error in the angular position of each wheel is considered as an input. Referring to Fig. 3, both the linear actuator forces (*F*_1_, *F*_2_) and the driving torques of the right and left wheels’ motors (*τ*_R_*, τ*_L_) are defined as inputs to the TWRM. In order to prevent any disturbance at the start of working as a result of lifting an object, since the TWRM is designed for the applications of picking and/or placing, two switching mechanisms are added to the system to insure the occurrence of system stability before proceeding with the object handling task and to prevent any disturbance that might affect the control effort. The mechanisms are designed in a way that the linear actuators will activate only when the TWRM’s IB reaches the stable upright position.

### BFO-PID control design

**Fig. 4:**
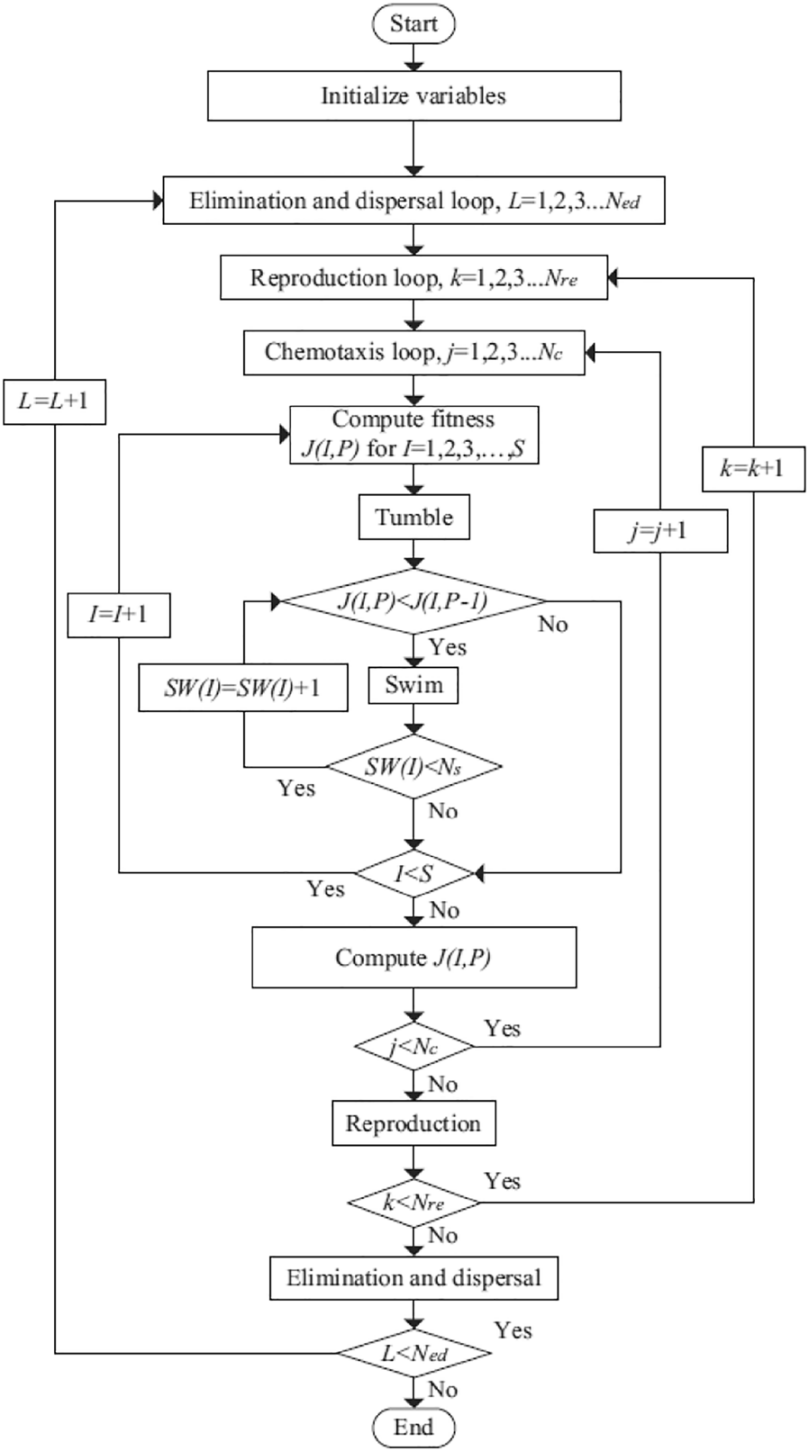
Flowchart of bacterial foraging optimization (BFO) [5]

This part deals with employing bacterial foraging optimization technique on the five-DOF TWRM’s PID control scheme, employed at earlier stages of this research, in order to control the vehicle by maintaining the TWRM’s IB in the upright position while counteracting the disturbances occurring due to various motion scenarios. The BFO main parameters are listed in Table 3, whereas Fig. 4 demonstrates the algorithm’s flowchart.

**Table 3:** BFO algorithm parameters [5]

Parameter symbol	Description
*p*	Search space dimension
S	Total number of bacteria in the population
*N*_s_	Number of bacteria swims in the same direction
*N*_c_	Number of chemotactic steps
*N*_re_	Number of reproduction steps
*N*_ed_	Number of elimination and dispersal events
*P*_ed_	Probability of the elimination and dispersal of bacterium
*C*	Step size of the bacterium tumble
*J*	Cost function value

In applying optimization techniques, the most crucial part is to select the objective functions that will be employed to evaluate the fitness function. Using performance indices to evaluate the controlled loops’ errors, the objective functions can be created. These performance indices, that have been utilized to optimize the system’s errors, can be defined as the following:

- Mean of the squared error (MSE).
- Integral of time multiplied by absolute error (ITAE).
- Integral of absolute magnitude of the error (IAE).
- Integral of the squared error (ISE).
- Integral of time multiplied by the squared error (ITSE).

**Fig. 5:**
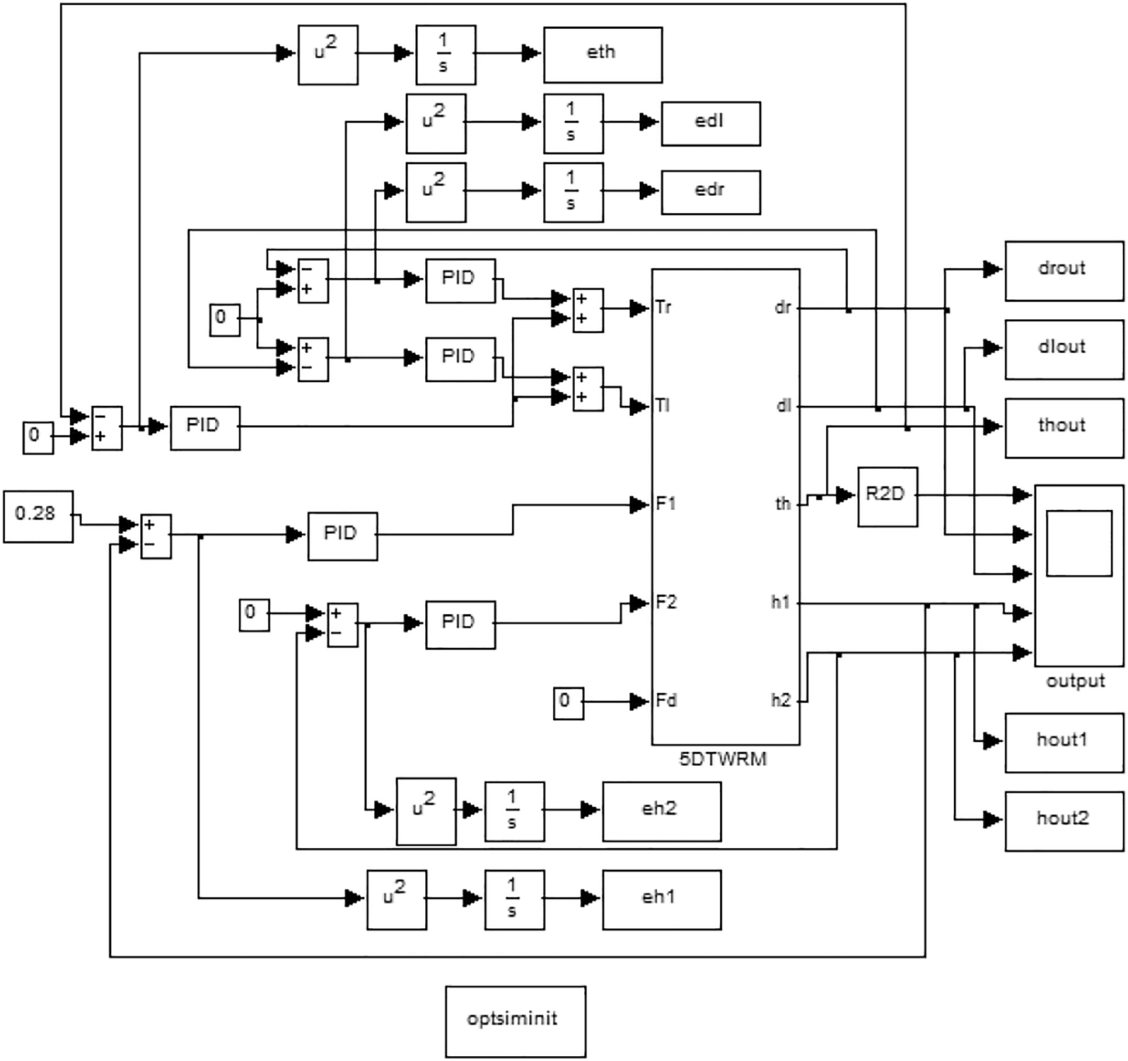
Simulink model of the PID controller optimized by BFO

Based on the study conducted by Goher and Fadlallah [25], the best optimized PID controller was the one optimized by IAE for the low percent overshoot and minimum settling time. MATLAB/Simulink model of the BFO-PID control method built to control the TWRM is illustrated in Fig. 5. Table 4 lists the controller gain parameters boundary limits for each of the five control loops that are implemented in MATLAB/Simulink environment with a view to optimize these gains.

**Table 4:** Controller gain parameters boundary limits

Controlled parameter	Gain parameter	Upper boundary	Lower boundary
*Loop 1*			
*δ*_R_	*Kp*_1_	20	− 20
	*Kd*_1_	20	− 20
	*Ki*_1_	0.1	− 0.1
*Loop 2*			
*δ*_L_	*Kp*_2_	20	− 20
	*Kd*_2_	20	− 20
	*Ki*_2_	0.1	− 0.1
*Loop 3*			
*θ*	*Kp*_3_	50	− 50
	*Kd*_3_	10	− 10
	*Ki*_3_	0.1	− 0.1
*Loop 4*			
*h*_1_	*Kp*_4_	20	− 20
	*Kd*_4_	10	− 10
	*Ki*_4_	0.1	− 0.1
*Loop 5*			
*h*_2_	*Kp*_5_	60	− 60
	*Kd*_5_	50	− 50
	*Ki*_5_	0.1	− 0.1

### PD-FLC control design

**Fig. 6:**
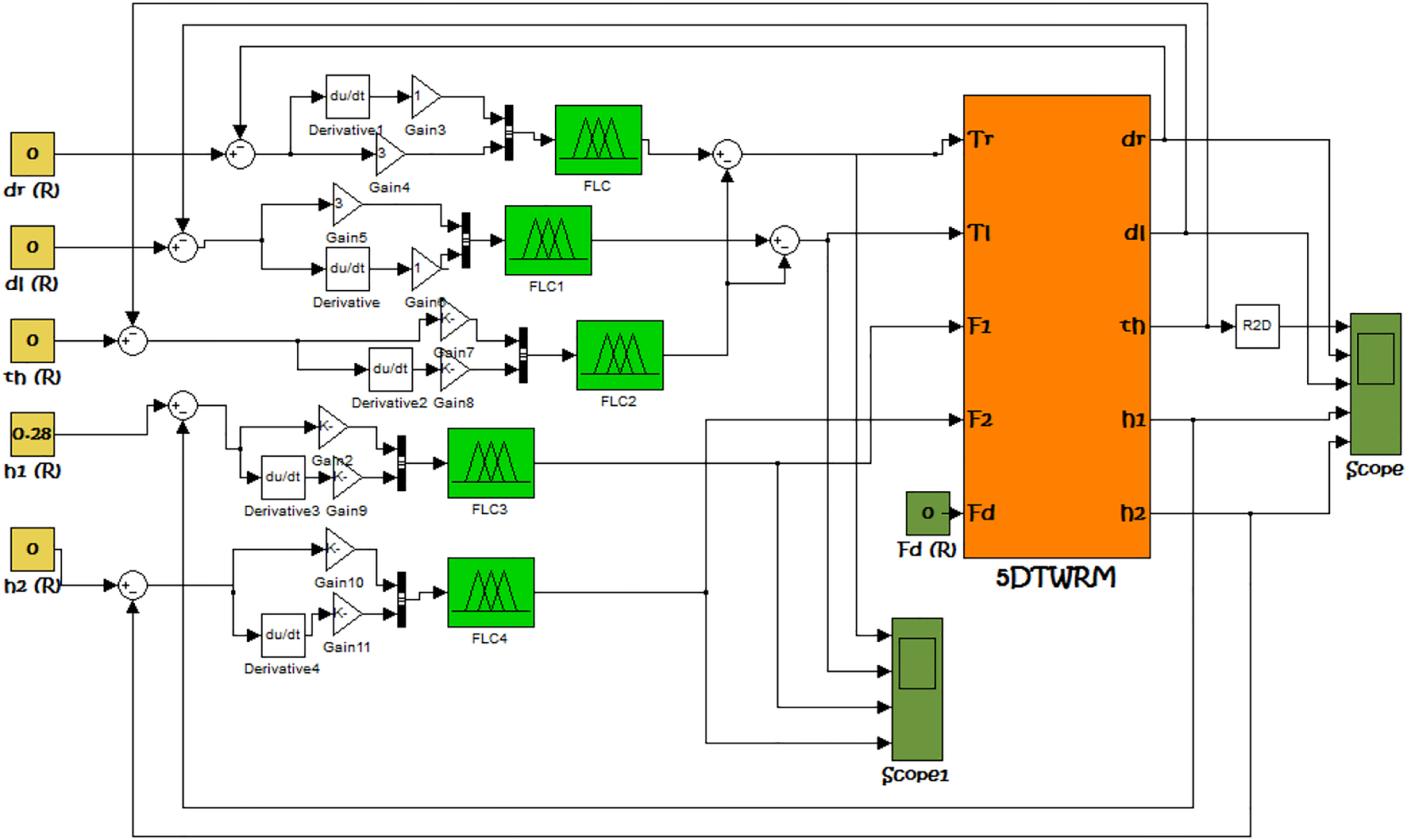
Simulink model of the FLC implementation

For the five-DOF TWRM, the author propose a control scheme that consists of a robust PD-like fuzzy logic control strategy (FLC), as demonstrated in Fig. 6, with five independent control loops designed to control the vehicle for multiple-motion scenarios. Simple Mamdani fuzzy approach are implemented in the control of the two-wheeled robotic machine, where the inputs are the angle and velocity and the output is multiplication factor. This factor will be multiplied with the potentiometer data and will affect the TWRM’s both right and left wheels’ velocity. The vehicle’s pitch angle and angular velocity feedback values are combined with fuzzy control, where the output is a multiplication factor that represents each wheel’s actuation values. Both the wheels’ angular velocity and the pitch angle consist of five membership functions. It is worth to mention that the steering system’s value will impact each wheel (left and right) independently but simultaneously. The multiplication factor consists of five membership functions from 0 to 1 [i.e., negative big (NB), negative small (NS), zero (Z), positive small (PS), and positive big (PB)]. The fuzzy output is multiplied with the steering value so it has two conditions for both right and left wheels. Each of the data will be combined in order to balance the vehicle’s IB while performing left and right turns. The total rules implemented to the five-DOF TWRM are listed in Table 5.

**Table 5:** Rules of navigation using fuzzy logic

Error	Change of error				
	NB	NS	Z	PS	PB
NB	NB	NB	NB	NS	Z
NS	NB	NB	NS	Z	PS
Z	NB	NS	Z	PS	PB
PS	NS	Z	PS	PB	PB
PB	Z	PS	PB	PB	PB

### Comparison between implementation of PID, BFO-PID, and PD-FLC

This section carries out a system response comparison, for various motion scenarios, between the three implemented control methods: PID controller, bacterial foraging-optimized PID controller, and PD-like fuzzy logic controller. Table 6 lists the control gain parameters utilized in each control loop for the three control methods with a view to attain a satisfactory system performance.

**Table 6:** Gain values for the three control schemes

Output parameter	Gain parameter	PID	BFO-PID	PD-FLC
*Loop 1*				
*δ*_R_	*Kp*_1_	80	10.255	7
	*Kd*_1_	75	0.016	3.5
	*Ki*_1_	0.05	15.05	0
*Loop 2*				
*δ*_L_	*Kp*_2_	80	10.255	7
	*Kd*_2_	75	0.016	3.5
	*Ki*_2_	0.05	15.05	0
*Loop 3*				
*θ*	*Kp*_3_	80	− 1.733	7
	*Kd*_3_	9	− 0.0693	1.5
	*Ki*_3_	0.02	0.0835	0
*Loop 4*				
*h*_1_	*Kp*_4_	8	10.3279	4.5
	*Kd*_4_	10	7.3378	6
	*Ki*_4_	0.01	0.013	0
*Loop 5*				
*h*_2_	*Kp*_5_	27	50.1502	14
	*Kd*_5_	32	30.7237	16
	*Ki*_5_	0.05	0.027	0

**Fig. 7:**
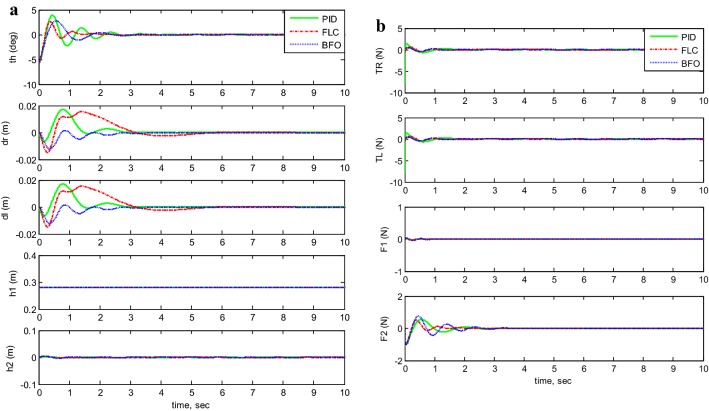
System output and input forces comparison for payload free movement (*h*_1_=* h*_2_=0). **a** System output and **b** system input forces

**Fig. 8:**
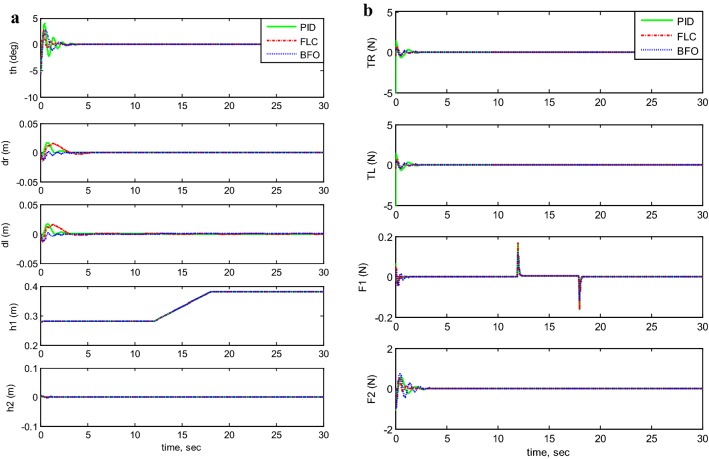
System output and input forces comparison for payload vertical movement only. **a** System output with moving *h*_1_ and **b** system input forces

**Fig. 9:**
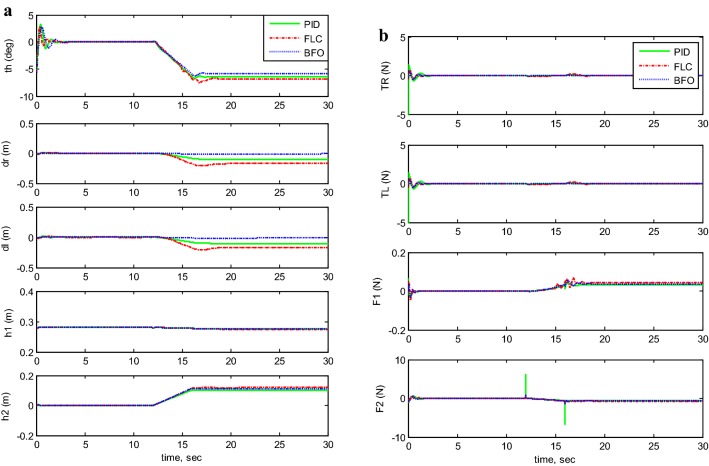
System output and input forces comparison for payload horizontal movement only. **a** System output with moving *h*_2_ and **b** system input forces

**Fig. 10:**
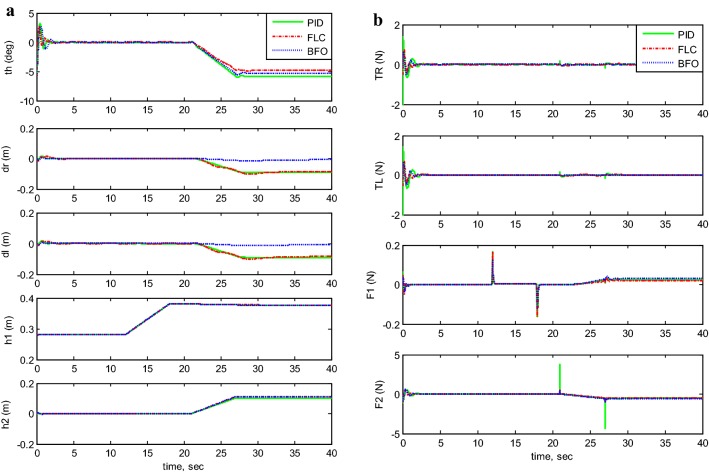
System output and input forces comparison for simultaneous vertical and horizontal motion (*h*_1_ and *h*_2_ ≠ 0). **a** System output with moving *h*_1_ and *h*_2_ and **b** system input forces

**Fig. 11:**
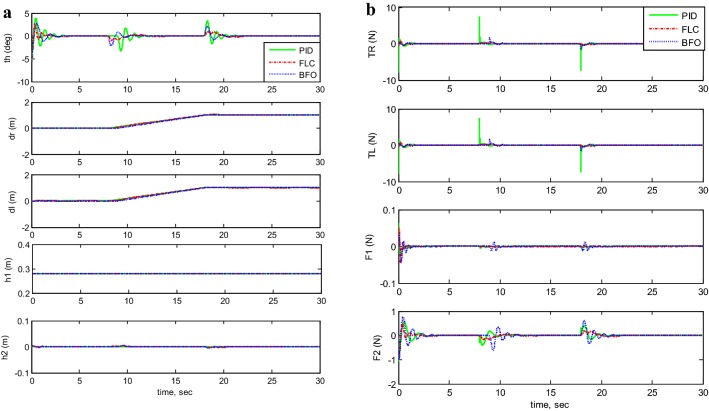
System output and input forces comparison for a 1-m straight line motion. **a** System output: straight line for 1 m, and **b** system input forces: straight line for 1 m

Figures 7, 8, 9, 10, and 11 illustrates the two-wheeled robotic machine mathematical model simulation output results, including the applied control effort, for five different case scenarios: payload free movement, payload vertical movement only, payload horizontal movement only, simultaneous horizontal and vertical motion, and 1-m straight line vehicle motion. As visualized in the previous figures, the BFO-PID control scheme has a superior performance and optimized behavior compared to the PID- and PD-like FLC control methods. It is also observable that the optimized controller by BFO algorithm reduces the applied input forces required to stabilize the robotic machine.

Taking the first motion scenario of payload free movement (*h*_1_ =* h*_2_ = 0) (Fig. 7) as an example, Table 7 lists a performance comparison between PID, PID-BFO, and PD-FLC control methods characterized by percentage overshoot, settling, rise, and peak times. Beginning with the system’s percentage overshoot, the PID controller optimized by bacterial foraging algorithm gives better overshoot value (27.9%), which is much lower than the recorded overshoot values for both PID- and PD-like FLC control schemes, 48.1% and 38.6%, respectively. As for the system’s settling time, the control strategy which is based on PID-BFO settles the vehicle in 0.78 s, which is three times less than the PID control method’s settling time (2.287 s) and two times less than the PD-FLC scheme (1.441 s). Moving to rise time values, the best result is given by PD-FLC (0.217 s), followed by BFO-PID method (0.23 s), and finally PID control scheme (0.2790 s). It can be seen that the rise time values are almost the same for all methods with small difference between them. As for peak time values, the PID controller has the highest peak value (0.5710 s), where the PD-FLC method value is the lowest but almost the same as the BFO scheme (0.4 s).

**Table 7:** System performance comparison between PID, PID-BFO, and PD-FLC control methods

Control method	Percent overshoot (%)	Settling time (s)	Rise time (s)	Peak time (s)
PID	48.1	2.2870	0.2790	0.5710
PID-BFO	27.9	0.7800	0.2300	0.4400
PD-FLC	38.6	1.4410	0.2170	0.4070

A phenomenon has been noticed in the scenarios of payload horizontal movement only case (Fig. 9) and the simultaneous horizontal and vertical motion case (Fig. 10). The TWRM’s stability was disturbed by the horizontal actuator’s activation, and the vehicle continues maneuvering instead of maintaining its initial position. This issue was only compensated by the BF-optimized PID controller, where it produced a satisfactory performance and robustness against the disturbance excited by the horizontal actuator’s activation.

#### Investigating real path trajectory with payload mass

**Fig. 12:**
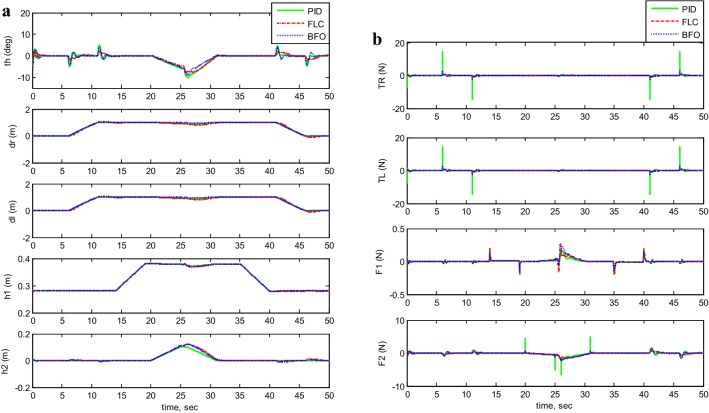
System output and input forces comparison for real path trajectory with payload mass. **a** System output: real path motion and **b** system input forces: real path motion

Since the TWRM is developed to be employed in industrial applications, Fig. 12 demonstrates the application where the robot will be used to manoeuver in a straight line and then activates both vertical and horizontal actuators in order to pick an object and return it to its initial position. As can be seen in Fig. 12a, the robot starts moving in straight line after achieving stabilization and the controllers act to maintain the robot’s stability. At the time the robot handles the load object, the stability of the system is not affected. Therefore, the controllers provide a good performance. Based on Fig. 12b, which represents the applied forces of the actuators, the PID control method consumes more forces than the forces applied by both BFO-PID and PD-FLC.

#### Control system robustness investigation

**Fig. 13:**
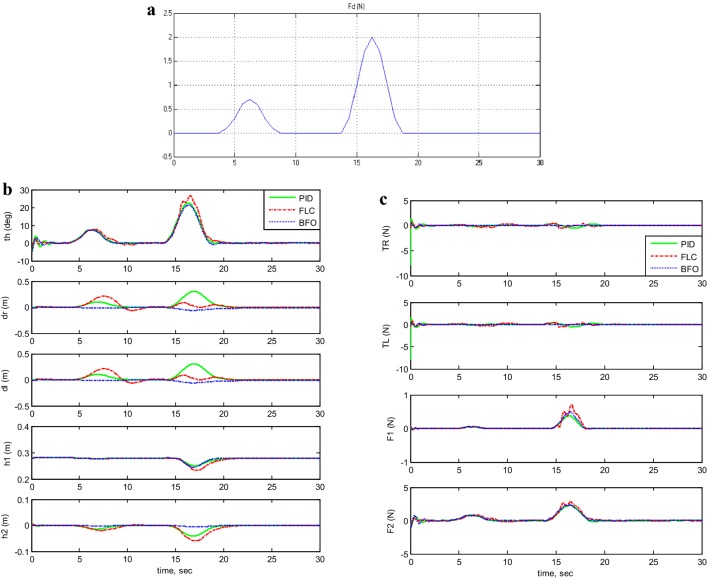
Response of system output and input forces by applying disturbance force. **a** Disturbance force applied, **b** the system output simulation with disturbance force, and **c** the system input forces of system with disturbance force

For the three proposed control methods, the TWRM stability was tested against the impact of disturbance force shown in Fig. 13a and the system performance is illustrated in Fig. 13b, c. As can be seen for the three control approaches, the vehicle in few seconds achieved its stability region about the vertical axis. However, the BF-optimized PID control method surpassed both PID and PD-FLC approaches in terms of withstanding the impact of disturbance on the vehicle wheels’ displacement (*δ*_R_, *δ*_L_) and the horizontal linear actuator displacement (*h*_2_). Therefore, in terms of robustness and instability minimization, BF-optimized PID control approach has a superior performance.

## Conclusions

Proportional–integral–derivative (PID) control scheme, bacterial foraging optimization (BFO) of PID control method, and fuzzy logic control (FLC) method have been applied on a novel five-DOF two-wheeled robotic machine (TWRM), and their performance has been compared in order to determine the optimum control strategy that provides the best stabilization performance for the system. The proposed TWRM’s nonlinear equations of motion have been derived using Lagrangian modeling approach and simulated with the assistance of MATLAB/Simulink^®^ environment. Based on the five case scenarios’ simulation results (i.e., payload free movement, payload vertical movement only, payload horizontal movement only, simultaneous horizontal and vertical motion, and 1-m straight line vehicle motion), the BFO-PID control scheme has a superior performance compared to the other two control methods. This performance has been reflected through the reduction in percent overshoot, rise time, and the applied input forces. The same performance was expected from the BFO-PID method when the system was tested against external disturbance forces. Despite the satisfactory performance of the system using BFO technique, BFA has a slow convergence speed and longer computation time which makes the implementation unrealistic in real-time tuning for solving a complex real-world problem. In this research, only simulation scenarios have been considered and hence little concern has been considered about the limitations of BFO. Future considerations of this work will consider implementing and comparing various optimization techniques such as genetic algorithm (GA), spiral dynamics (SD), hybrid spiral dynamics bacterial chemotaxis (HSDBC), and particle swarm optimization algorithm (PSO) for optimizing the TWRM’s PID controller gains in order to improve the system’s stabilization performance. Furthermore, investigating the robustness of the system will be considered not only in the application scenario, but also in the system itself. By changing the system’s physical parametric specifications, the performance of the proposed control methods in different parameters of the system will be evaluated.

Moreover, the TWRM’s hardware model can be built and the performance of the control approaches implemented on the system will be examined against real disturbance forces for real industrial applications.
